# Classifying and inferring behaviors using real‐time acceleration biotelemetry in reproductive steelhead trout (*Oncorhynchus mykiss*)

**DOI:** 10.1002/ece3.5634

**Published:** 2019-09-27

**Authors:** Nathaniel T. Fuchs, Christopher C. Caudill

**Affiliations:** ^1^ Department of Fish and Wildlife Sciences College of Natural Resources University of Idaho Moscow ID USA; ^2^ Present address: Washington Department of Fish and Wildlife Twisp WA USA

**Keywords:** accelerometer, biotelemetry, intragastric, radio telemetry, steelhead spawning

## Abstract

Movement behaviors are central to ecology and conservation. Movement sensing technologies can monitor behaviors that are otherwise difficult to observe under field conditions and may enhance the ability to quantify behaviors at the population scale. We monitored steelhead trout (*Oncorhynchus mykiss*) spawning behaviors in a seminatural enclosure using accelerometer telemetry tags while simultaneously observing behaviors with underwater cameras. Behavioral assignments from visual observations were compared to acceleration histories to develop assignment criteria for acceleration data, including for a key behavior (oviposition). Behavioral events independently classified using acceleration data prior to reviewing video were compared to video scoring and 97% of holding behaviors, 93% of digging behaviors, and 86% of oviposition/covering behaviors were correctly assigned using acceleration data alone. We applied the method to *at‐liberty* steelhead in spawning tributaries. Acceleration records revealed putative spawning and oviposition in *at‐liberty* female steelhead, and time budgets for *at‐liberty* steelhead were similar to those monitored within enclosures. The use of similar movement sensing tags and classification approaches offers a method for monitoring movement behavior, activity budgets, and habitat use in a broad array of aquatic and terrestrial taxa, and may be especially useful when behaviors are cryptic.

## Graphical abstract

Movement behaviors are central to ecology and conservation, and movement sensing technologies can monitor behaviors that are otherwise difficult to observe under field conditions and may enhance the ability to quantify behaviors at the population scale. We monitored steelhead trout spawning behaviors in a seminatural enclosure and in the natural environment (*at‐liberty*) using accelerometer telemetry tags while simultaneously observing behaviors with underwater cameras. Behavioral events independently classified using acceleration data prior to reviewing video were compared, and 97% of holding behaviors, 93% of digging behaviors, and 86% of oviposition/covering behaviors were correctly assigned for steelhead observed within the enclosure. Applying the same methods to *at‐liberty* steelhead, acceleration detections revealed putative spawning and oviposition behaviors.

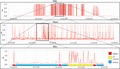

## 1 INTRODUCTION

Movement behaviors of animals are linked to individual fitness at multiple scales. Therefore, understanding movement behavior is critical to understanding factors affecting species of ecological and management interest. Collecting movement data on species in visually limited environments has been particularly challenging. Improvements in optics (Graham, Jones, & Reid, 2004), acoustic cameras (Martignac, Daroux, Bagliniere, Ombredane, & Guillard, 2015; Mueller, Brown, Hop, & Moulton, 2006), and the use of passive integrated transponder (PIT) tag (Roussel, Haro, & Cunjak, 2000) and accelerometer tag technology have enhanced the capacity for tracking movements and behaviors (Broell, Taylor, Litvak, Bezanson, & Taggart, 2016; Moser, Corbett, Burke, & Langness, 2018; Thiem et al., 2015; Watanabe, Wei, Du, Li, & Miyazaki, 2013). The development of commercial accelerometer telemetry tags will provide opportunity to detail behaviors in situ for many species. A growing body of research has begun applying new acceleration sensing technologies with the aim of better understanding habitat preference, migratory, and foraging behavior in many species (Laich, Wilson, Quintana, & Shepard, 2008; Wakefield, Phillips, & Matthiopoulos, 2009; Wang et al., 2015; Weegman et al., 2017). Determining the relationship between specific behaviors and acceleration time series data is a key step in implementing accelerometer tag technology in field studies of *at‐liberty* animals.

Pacific Salmon and trout (*Oncorhynchus* spp.) provide important ecological, economic, recreational, and societal benefits. Consequently, declines in many populations have raised concerns among fisheries managers (Nehlsen, Williams, & Lichatowich, 1991). Spawning behaviors strongly affect lifetime fitness in anadromous salmonids because many species are semelparous or nearly so. Steelhead (anadromous Rainbow Trout, *O. mykiss*) are frequently iteroparous in coastal populations, but are nearly functionally semelparous in interior populations migrating upstream 100s of km prior to spawning (iteroparity <5%; Keefer et al., 2017; Keefer, Wertheimer, Evans, Boggs, & Peery, 2008; Leider, Chilcote, & Loch, 1986). Summer‐run steelhead populations enter freshwater in late summer (July–September) and overwinter in freshwater before spawning during spring months (March–May). Consequently, migration success, holding success (survival during overwintering), and spawning success (successful redd building and egg deposition) are important life history parameters.

Anadromous salmonids reproduce in freshwater after return from the ocean, where the female selects a site in the natal stream to dig a nest (redd) and deposit eggs while males fertilize them (Esteve, 2005; Fleming, 1998; Quinn, 1999). Specific spawning behaviors (i.e., digging, covering, oviposition; Needham & Taft, 1934; Orcutt, Pulliam, & Arp, 1968; Shapovalov & Taft, 1954; Tautz & Groot, 1975) are consistent across taxa and populations, including populations of steelhead, and include the following sequence. Adult female steelhead select a redd location with suitable flow conditions and clear fine sediments by beating their tails against the substrate (digging). Once constructed, the female “probes” the substrate with her anal fin prior to depositing eggs. Males position themselves alongside the female while “quivering” (hereafter “coaxing”) and release milt as she deposits eggs (oviposition). The female immediately covers the eggs with substrate by frequent and rapid tail beats. Additional digging/oviposition events occur until the female has released all her eggs.

While there is rich literature detailing salmonid spawning behaviors, quantifying spawning behavior is challenging for steelhead as they spawn in spring when elevated flows and high turbidity are common. Fisheries managers often rely on redd counts to estimate spawning escapement, and spawning success at the population level (Zimmerman & Reeves, 2000), but low visibility, potential interpopulation differences in redd digging and oviposition behavior, and limited redd detection duration introduce substantial uncertainty (Esteve, 2005; Gallagher & Gallagher, 2005). While radio telemetry can be used to evaluate migration behavior, habitat use, and survival in juvenile and adult anadromous fishes from local (10–100 m) to large (0.1 to >1,000 km) scales (Boggs, Keefer, Peery, Bjornn, & Stuehrenberg, 2004; Cooke et al., 2006; Keefer, Peery, Bjornn, Jepson, & Stuehrenberg, 2004; Keefer, Peery, & District, 2008; Keefer, Caudill, Peery, & Moser, 2013), the typical resolution of radio telemetry tags (~10–25 m) has been insufficient to discriminate among some behaviors, including those exhibited during spawning (Heim, Steeves, McMahon, Ertel, & Koel, 2018).

Accelerometry provides instrumentation to address such issues. Accelerometry measures acceleration—the rate of change in velocity of objects along one or more axis of movement, often reported in units of standard gravity (1 gravity [g] = 9.807 m/s^2^). Tsuda et al. (2006) used archival acceleration data loggers surgically attached to the backs of chum Salmon (*Oncorhynchus keta*) that monitored the amplitude of 2‐dimensional surge and swaying acceleration. Data were accessible only after loggers were recovered after the monitored fish had spawned and died. The development of acceleration telemetry (i.e., accelerometers transmitting data in real time) provides the opportunity to quantify behavior without tag recovery. Advances in analytical approaches are also needed for emerging telemetry data.

**Figure 1:**
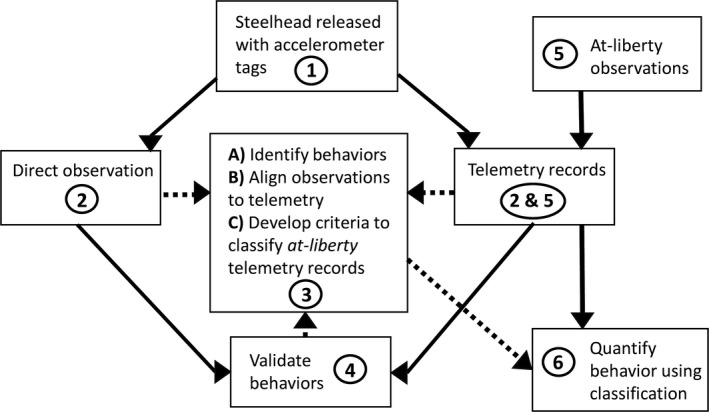
Overview of study design and method: (1) tag and release steelhead with accelerometer tags for observation within an enclosed monitoring space; (2) simultaneously record behaviors via underwater video camera and monitor acceleration; (3) identify behaviors from video observations, align observed behaviors to accelerometer time series, and establish criteria for classifying acceleration records using the amplitude, frequency, and variability of the acceleration time series; (4) comparison of video and telemetry‐classified behaviors using independent subsets of the data; (5) tag, release, and monitor a sample of *at‐liberty* steelhead; and (6) classify acceleration histories of *at‐liberty* animals to quantify key behaviors

The goal of this research was to quantify behaviors in spawning adult steelhead using acceleration data transmitted from tagged fish spawning in the wild after associating patterns of acceleration to specific behaviors in a controlled spawning enclosure (Figure 1). Our objectives for the enclosure samples were to (a) video record behaviors and simultaneously monitor the associated tag acceleration; (b) classify the recorded behaviors and align them to accelerometer times series; (c) establish criteria for identifying each behavior solely from the accelerometer records using amplitude, frequency, and variability; and (d) validate the telemetry‐classified behaviors using unviewed behavioral observations from the enclosure by testing for differences in individual time budgets estimated from video versus inferred from acceleration records. Our goal for the *at‐liberty* steelhead was to classify behaviors using the criteria developed in the enclosures, focusing on female oviposition events.

## 2 MATERIALS AND METHODS

### 2.1 Accelerometer tags

Steelhead were monitored using prototypes of a commercial 3‐volt coded transmitter (model MCFT3‐3A, Lotek Wireless; 16 mm × 58 mm; 20 g in air; estimated tag life of 90–120 days). The prototype tags measured acceleration at 12.5 Hz and transmitted the tag code and the maximum differential acceleration every four seconds, referred to as transmission burst interval (BI). The maximum differential acceleration (hereafter, acceleration) was the maximum acceleration in any of three axes during the 4‐s sampling interval, with a maximum 1.5 gravity (g) acceleration and 0.03 g resolution. Tags were tested and activated asynchronously to minimize transmission collisions. Telemetry data were recorded using Lotek SRX800 Receivers and aerial 4‐element Yagi antennas using standard radio telemetry methods. Qualitative range testing revealed similar detection distances to that of standard radio tags and was 100–150 m using 4‐element antennas.

### 2.2 Enclosure observations

**Figure 2:**
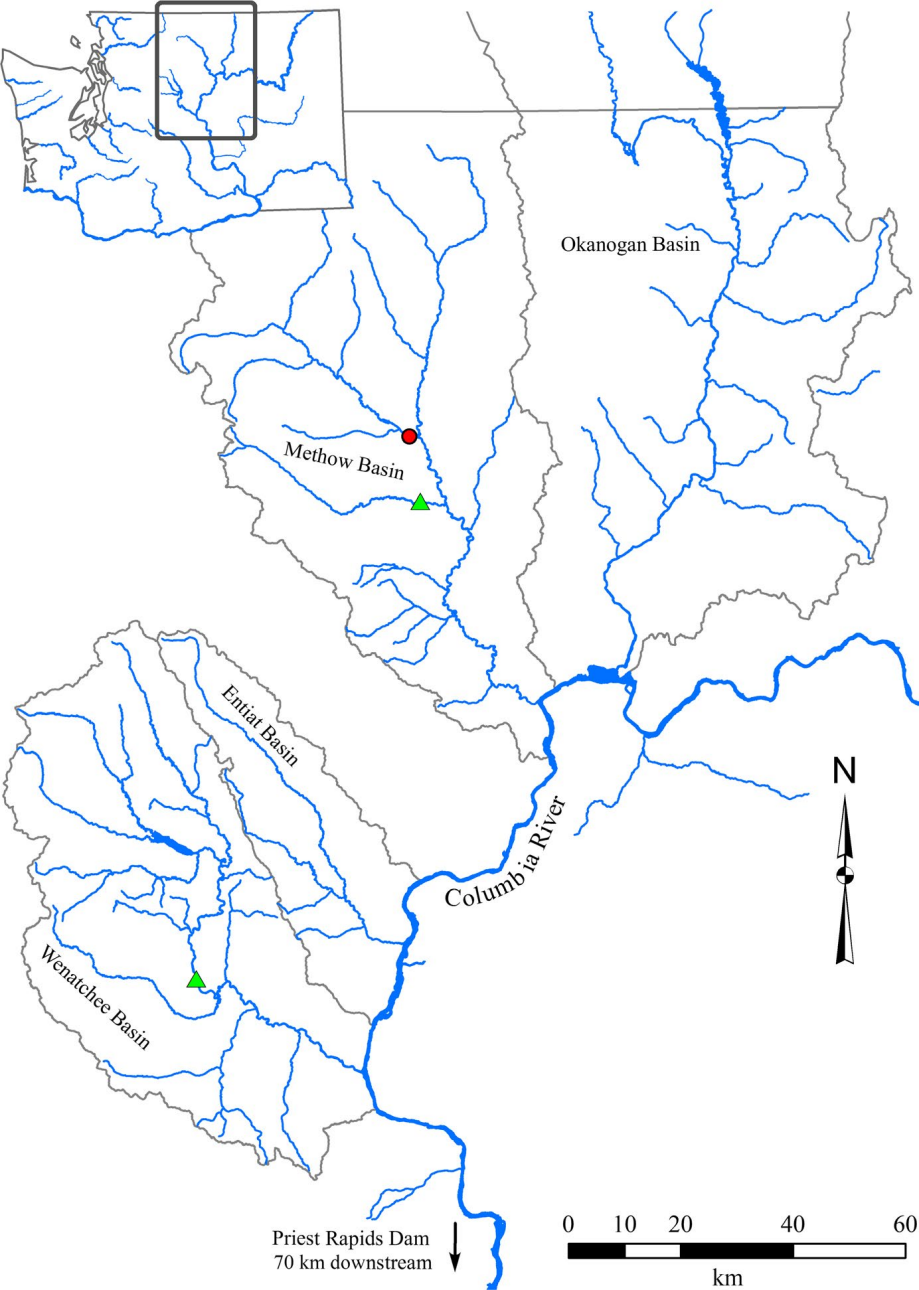
Upper Columbia River basin study site locations. The enclosure experiment took place at Winthrop National Fish Hatchery spring creek acclimation site (red dot). Tagged fish release sites were Tumwater Dam (Wenatchee River) and Twisp River weir in the Methow River basin (green triangles)

We observed spawning using optical video under seminatural conditions at Winthrop National Fish Hatchery (WNFH) on the Methow River (North central Washington State, USA), a major tributary to the Upper Columbia River basin (Figure 2). Steelhead returning to WNFH must migrate >920 km upstream, passing nine major hydro‐electric dams. Each steelhead received an intragastrically implanted accelerometer tag and passive integrated transponder (PIT) tag implanted ventrally between the pelvic fins. A band of surgical rubber tubing placed around the accelerometer was used to reduce regurgitation (Keefer, Peery, Ringe, & Bjornn, 2004; Thorstad, Rikardsen, Alp, & Økland, 2013). Fish were hatchery‐origin and were collected, anesthetized, tagged, and released by USFWS Mid‐Columbia Fish & Wildlife Conservation Office staff. Fish were collected at the hatchery after entering a volitional ladder trap or were caught using hook and line methods by hatchery staff. Two groups of tagged fish were observed, and both groups consisted of two males and two females. Females were 68–74 cm, males 55–74 cm, and recording duration was 24–38 hr per group. Males and females were tagged exclusively on two different frequencies in order to minimize the collision of transmissions on a given frequency.

A 12 m long, 2–4 m wide temporary enclosure was created in a diversion channel. Stainless steel weir panels bounded the downstream of the enclosure and two screened 0.7 m diameter culverts bounded the upstream of the enclosure and also provided cover within the culverts and under the culvert outflow. Spawning gravel was present throughout the enclosure (gravel [2–64 mm] to cobble [64–256 mm]) and depth ranged from 0.5 to 1.0 m. Anadromous steelhead had been observed by USFWS spawning naturally in the outflow channel in a year when the barrier structure failed.

Fish movements and behaviors were monitored and recorded using 4 SPECO underwater cameras (Global Equipment Company Inc.) while acceleration data were recorded by receivers operating continuously and positioned on site within 20 m of the enclosure. The first sample group was monitored by video from 26 April to 28 April 2017 and thereafter by telemetry receiver until 1 May 2017. The second sample group was monitored from 3 May to 5 May 2017 until a high flow event resulted in poor visibility (beginning 4 May) and two fish (M4 and F4) escaped the enclosure. The latter fish were not evaluated for behavior in the enclosure but were considered *at‐large* after escape on the evening of 4 May 2017.

### 2.3 Behavior in enclosures

All tagged females in each group that remained within the enclosure were observed on video exhibiting spawning behaviors (digging, oviposition/covering) within two days of release. Episodic digging lasted several hours followed by several hours of inactivity (based on telemetry records and visual observations). Based on available literature and preliminary review of data, the following behaviors were identified:

#### 2.3.1 Holding

Holding was defined as a fish remaining in place in the water column or moving <1 body length/BI (Figure 4a).

#### 2.3.2 Digging

Digging was assigned when a female was observed rolling onto one side and beating her tail rapidly against the stream substrate dislodging rocks and gravel for a span of 1.0–2.5 s (Figure 4b).

Each event had two to seven tail beats and frequently carried the female upstream. The female would immediately resume an upright position and return to her original location on the redd by swimming downstream in a loop pattern (scored as “lateral movement/looping”) or allowing the current to carry her downstream while remaining oriented upstream.

#### 2.3.3 Oviposition and covering

At the onset of oviposition, females pitched their tail downward into the substrate and released eggs while an adjacent male released milt. During oviposition, all fish opened their mouths wide and quivered their bodies slightly for a duration of ~6 s (1–2 BI). Oviposition events were immediately followed by a series of covering events (Figure 4c), each similar to digging events, but with increased and more regular frequency (4–5 events/min) and slightly shorter duration.

#### 2.3.4 Lateral movements

Lateral movement was assigned when a fish was observed moving laterally with a return to its original position within the time span of a single BI (4 s). The most common lateral movements were females on redds moving side‐to‐side between specific digging events. In some cases, the female looped back to her original position immediately following digging (looping). Lateral movements were common among males when in close proximity to a female, and included some up‐ and downstream movements (Figure 4d).

#### 2.3.5 Aggression

Aggression among fish within the spawning enclosure occurred between fish of the same sex and usually involved a larger fish charging a smaller fish. Typically, the largest male in the enclosure would repeatedly attack a smaller male, which would in turn attack untagged precocial juvenile male steelhead (not excluded from entering the enclosure through the weir panels).

#### 2.3.6 Burst movements

Burst movements were classified as rapid swimming events resulting in at least one body length movement per BI. Burst movements were typically longitudinal (up/downstream) and less than one BI in duration (Figure 4f).

#### 2.3.7 Coaxing

Coaxing was assigned when a male positioned alongside a female on redd, ran his nose along the female's side and, holding position, rapidly undulated alongside the female for 1–2 s (Figure 4g). All males were observed coaxing.

#### 2.3.8 Out of view

“Out of view” was scored when a fish was not visible on any camera within the enclosure. Out‐of‐view events occurred following a significant burst or lateral movement, during aggressive interactions, and during the first ~1 hr following release of fish into the enclosure. Out‐of‐view periods were excluded when developing criteria for accelerometer records.

### 2.4 Development of classification criteria for accelerometer records

Behaviors were manually scored from video for the telemetered steelhead. Randomly selected 10‐ to 15‐min periods from each clock hour of daytime video were reviewed for each steelhead. Start and end times for behaviors were assigned for the entire period. Observations were limited to daylight hours (~6:00 a.m. to 8:00 p.m.) due to limited visibility from dusk to dawn. Behaviors were identified and cataloged by a single experienced reviewer (Fuchs) using preliminary video observations and existing literature (Esteve, 2005; Needham & Taft, 1934; Orcutt et al., 1968; Shapovalov & Taft, 1954; see Section 3). Behavioral events observed in the video records were identified to estimate individual time budgets, and the start and end time of each event was collated as time stamps. These time stamps were then aligned to accelerometer records. We examined the distributions of acceleration records for each behavior observed in the video recordings to identify thresholds and decision rules for assigning behaviors based on the amplitude, frequency, and variability of acceleration records. An independent subset of the telemetry records were reserved to assess accuracy of behavior by comparing behavioral assignments from video after assigning behaviors using only acceleration criteria for female behaviors (the focus of the study).

### 2.5 Biotelemetry of at‐liberty steelhead

Steelhead were tagged, released, and monitored in the Twisp and Wenatchee Rivers in north/central Washington State (USA; Figure 2). Two females and one male were released at Twisp River weir (~75 km up the Methow River) in April–May of 2016, and four females and two males were released at Tumwater Dam (~45 km up the Wenatchee River) in April–May of 2017. Steelhead were tagged as before with the exception that tags transmitted every 3, 3.5, or 4 s to minimize potential for continuous transmission collisions between tags.

Released fish were monitored for movement using mobile tracking and PIT records until they left the system or were classified as regurgitated tag/dead based on continuous zero g acceleration records. Steelhead were mobile tracked until observed at a presumed spawning location (i.e., at riffles, or were detected at historic known spawning sites) for 24 hr, after which a fixed site receiver and antenna were installed near to their detection location. Fixed sites were visited daily, and fish presence, antenna placement, and battery life were verified until the fish was detected leaving the location. *A*cceleration records were classified using criteria from the enclosure steelhead, and time budgets were generated for *at‐liberty* steelhead.

### 2.6 Statistical analysis

A Kruskal–Wallis test followed by a post hoc Dunn's test was used to test whether mean acceleration values differed among behaviors assigned using video obtained in the enclosures. We used a chi‐square test to test whether the frequency of behavioral events assigned during validation using video observations differed from frequencies inferred using acceleration records across individual steelhead. Statistical analyses were implemented using R version (R core team 2018).

## 3 RESULTS

**Figure 3:**
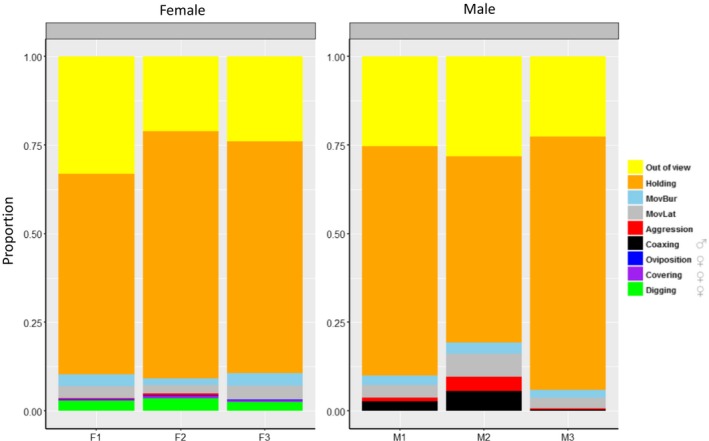
Proportion of time tagged fish were observed displaying each behavior during video monitoring. Coaxing behavior observations (red) pertain to males only while oviposition (dark blue), covering (violet), and digging (green) were exclusive to females only

Based on video observations, steelhead of both sexes spent the majority of time holding (mean = 63%; Figure 3) in the enclosures, while other nonspawning movement behaviors combined (burst movement, lateral movement, and aggression) comprised a mean ~ 7% of the total observed time budget. Spawning‐related behaviors (digging, oviposition/covering, and coaxing) accounted for ~6% of observed behaviors, and the remaining ~25% of time was spent out of view.

**Figure 4:**
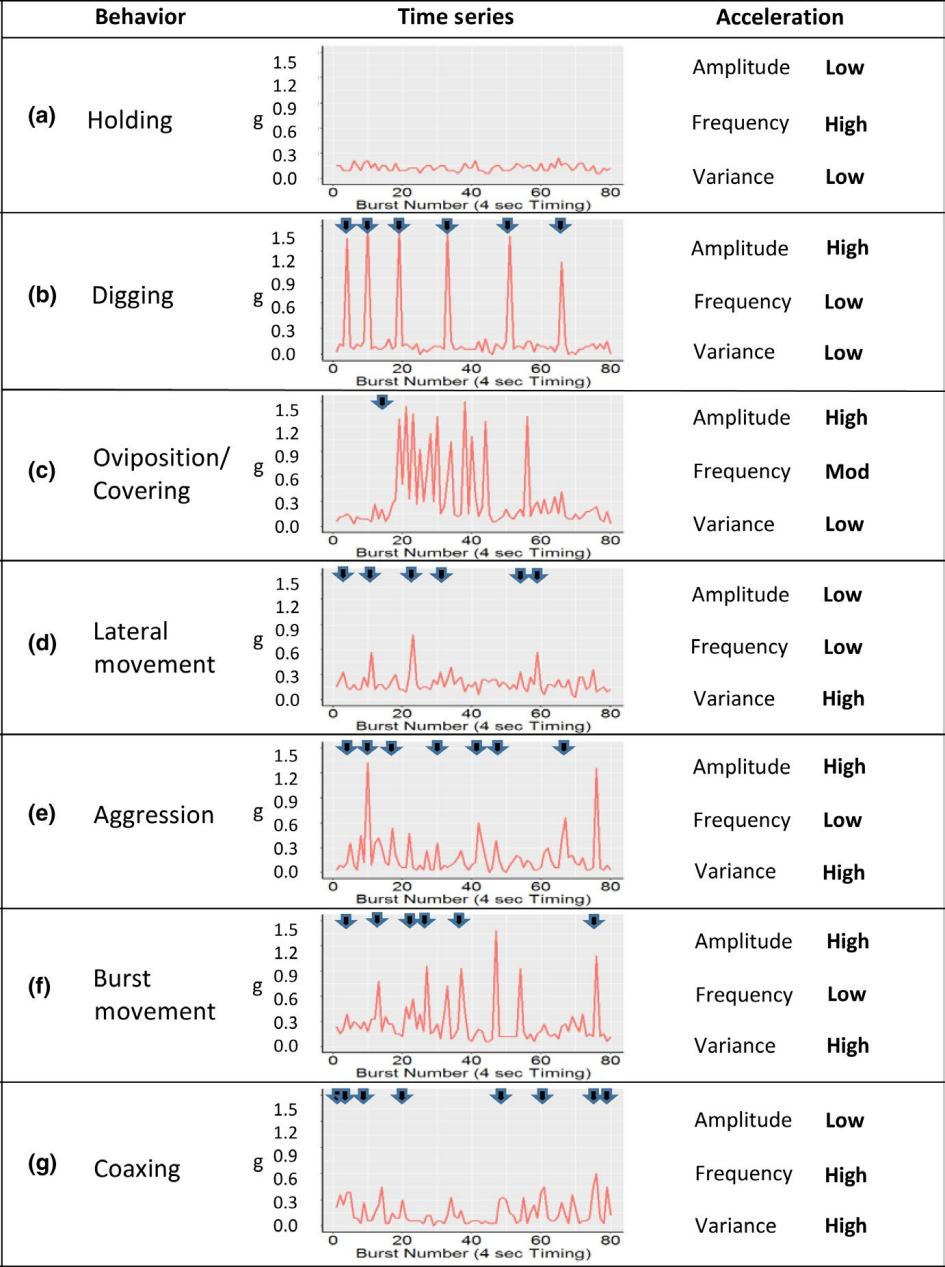
Acceleration time series for behaviors identified during video observation of steelhead in the spawning enclosure. Tag transmission interval was 4 s, and each series represents approximately 5 min. The beginning of each behavioral event observed by video is indicated by black arrows

**Figure 5:**
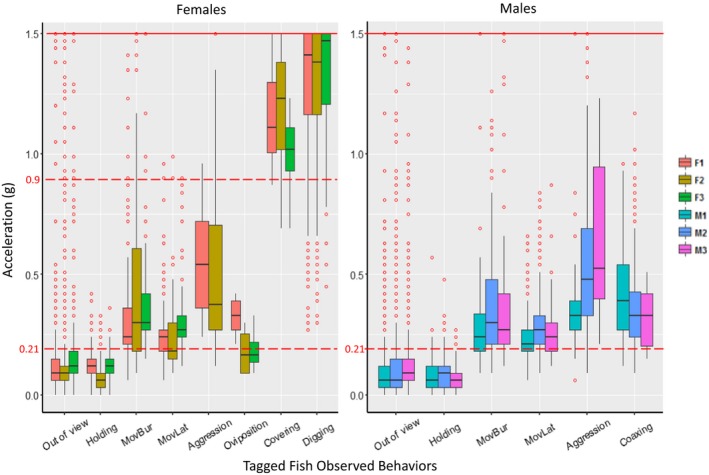
Distribution of acceleration (g) recorded during each behavior observed by video in spawning enclosures. Distributions separated by sex and each detection of acceleration represents a single burst interval. Upper horizontal red line indicates maximum acceleration recordable by the tag, and dashed lines indicate selected spawning detection thresholds for digging and covering (0.9 g) and holding (0.21 g)

Acceleration records differed in magnitude, frequency, and variance among behaviors (Figures 4, 5). Holding behaviors were most frequently observed when fish were away from a redd, and preceding and immediately following a digging event. Holding acceleration rarely exceeded 0.3 g, and long duration holding periods were common. Digging events were <4 s duration (mean = 1.9 s), and thus most were recorded with a single BI record, with some events recorded by two records. Digging behavior acceleration averaged 1.26 g (range: 0.8–1.5 g). Acceleration during oviposition was low (mean = 0.24 g, range 0.06–0.42 g) and was always immediately followed by a series of higher acceleration covering events (Figure 4c). The duration of a single covering event rarely exceeded 1.5 s (mean = 1.2). Average acceleration detected during covering was 1.15 g (range: 0.7–1.5 g) and each was followed by intervals of low acceleration lasting 5–30 s. Postoviposition covering typically lasted between 2–6 min and averaged 14 consecutive covering events. Acceleration during lateral movements was low/moderate and averaged 0.27 g (range: 0.15–0.5 g). Aggressive chasing behaviors by dominant males were commonly observed in an apparent attempt to exclude access to females. Aggressive movements were most often <4 s (1 BI) though some were sustained for 16–20 s (4–5 BI). Aggression between females was less prevalent. Acceleration during aggression events averaged 0.51 g (range: 0.21–1.5 g). Burst movement acceleration averaged 0.3 g and ranged from 0.15 to 0.75 g with occasional acceleration records reaching the maximum accelerometer threshold of 1.5 g. Coaxing was most common just prior to oviposition, after which males would typically hold behind the female until covering events ceased. Acceleration during coaxing was moderate and averaged 0.37 g (range: 0.15–0.8 g).

### 3.1 Development of classification criteria for accelerometer records

**Figure 6:**
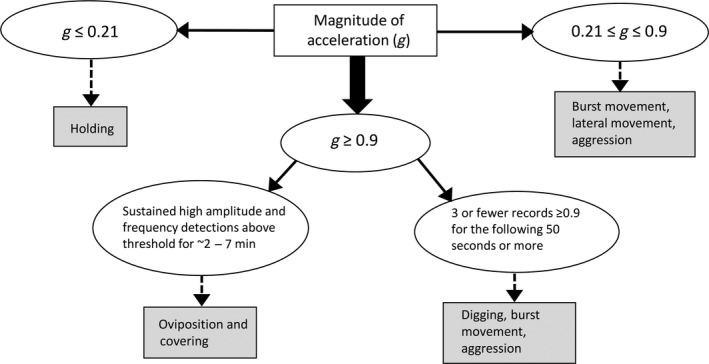
Stepwise criteria for assigning behaviors to acceleration records. Acceleration thresholds (g) represent the magnitude of acceleration detected for each BI above or below the specified thresholds

Distributions of acceleration records differed among some, but not all behaviors (Figure 5) and were used to develop decision rules for classification (Figure 6). Notably, sets of behavior were largely distinct based on the magnitude of acceleration alone. The mean acceleration during holding was 0.09 g across sexes (0.08 g for males, 0.10 g for females; see Table A1 for results for individual steelhead). Acceleration records of 0.0 g (no movement) were common (4.0% of holding behavior detections), though durations of no movement >3 consecutive BIs were exceedingly rare (0.02% of total time).

A large majority of digging and covering behaviors were associated with acceleration exceeding a 0.9 g threshold. The average acceleration for digging events was 1.27 g and 88% exceeded a 0.9 g threshold (*n* = 422 events); 41% were at the maximum recordable acceleration (1.5 g). Mean covering event acceleration was 1.14 g, and 93% (*n* = 76) exceeded 0.9 g. A small percentage of aggression (10%) and burst/lateral events (6%) had >0.9 g. Only 1.5% of coaxing events exceeded 0.9 g. Notably, 94% of out‐of‐view observations registered at or below the 0.21 g holding threshold, suggesting holding was the dominant behavior during those periods. Mean acceleration was slightly higher for females than males (0.60 g and 0.52 g, respectively), and one female (F3) was never observed displaying aggression. Aggressive behaviors (mean of 0.55 g) were more frequent in males and included the highest accelerations recorded for males (see Table A2). Male‐to‐male aggression was recorded on all days of observation. Results of the Kruskal–Wallis test indicated significant differences in acceleration (*X* ^2^ = 6,718.4, *p* = 2.2*10^‐16^) among several of the observed behaviors (Table A3).

While high magnitude acceleration events (>0.9 g) were associated with several behaviors and not all behaviors could be distinguished using mean acceleration, oviposition/covering could be reliably separated from digging/burst movement/aggression events because covering events were recognizable from relative high frequency events >0.9 g (mean ~ 5 events/min) spanning 2–7 min, whereas digging records >0.9 g were lower frequency and less regular (e.g., compare Figure 4b with 4c). Oviposition events were indistinguishable from holding events based on acceleration during oviposition (mean of 0.24 g), but oviposition could easily be identified indirectly by the frequent covering events following oviposition (Figure 4c). An average of 14 covering events (range 9–24) occurred approximately every 8–12 s after oviposition for a mean duration of 3:26 (min:seconds; range 1:43–6:19). Overall, the combined criteria distinguished among three behavioral classes: holding, oviposition/covering, and digging/burst movement/aggression (Figure 6).

Validation using an independent subset of records from the enclosure indicated >92% correct classification of digging and holding events (Table 1). Burst and lateral movement behaviors were correctly assigned less consistently (48% and 45%, respectively). Oviposition events were correctly assigned in ~ 86% of the small sample of events in the subset (*n* = 7). The overall frequencies of behaviors assigned using video observations versus acceleration records did not differ in the validation samples (chi‐square, *p* = 0.63).

**Table 1:** Counts and proportions (by row, in parenthesis) of behaviors assigned using accelerometer records and observed behaviors using video for females during validation (*N* = 3)

Inferred behavior	Observed behavior					Total
	Digging	Holding	Burst moves	Lat moves	Oviposition/covering	
Digging	**264 (0.93)**	0	14 (0.05)	7 (0.02)	0	285
Holding	1 (<0.01)	**2,089 (0.97)**	15 (<0.01)	58 (0.02)	0	2,163
Burst moves	8 (0.05)	39 (0.22)	**83 (0.48)**	43 (0.25)	0	173
Lat moves	2 (0.01)	62 (0.31)	42 (0.21)	**95 (0.47)**	0	201
Oviposition/covering	0	0	1 (0.14)	0	**6 (0.86)**	7

Note

Counts and proportions correctly assigned are indicated in bold.

### 3.2 Biotelemetry of at‐liberty steelhead

In total, 7 of 11 *at‐liberty* released steelhead (5 females and 2 males) had suitable detection histories for inferring behaviors (Table 2). Three females had records classified as oviposition, with 3–6 events per female and a daily oviposition rate of ~0.6 events per day (mean monitoring duration = 8 days per female; range 5–11 days). *At‐liberty* males were difficult to monitor continuously because they rarely remained at any one location for longer than a single day. Four of the tagged fish were presumed to have died/shed their tags within one week of release.

**Table 2:** Size, sex, release locations and dates, approximate detection durations, and presumed fates after release for *at‐liberty* steelhead

Tag ID	Sex	FKL (cm)	Release site	Release date	Days	Presumed behaviors and fates
I16	Ma	74	Twisp	3 May 2016	6	Active 3 days, shed tag/mortality
I19	Fe	60	Twisp	29 April 2016	9	Fallback below weir, active
I24	Fe	61	Twisp	2 May 2016	10	Detected spawning, kelted
I26	Fe	72	Tumwater	15 April 2017	2	Entered unmonitored tributary, kelted
I27	Ma	73	Tumwater	17 April 2017	~1	Fallback below dam
I21	Fe	77	Tumwater	20 April 2017	~1	Shed tag/mortality, 1 day after release
I22	Fe	77	Tumwater	20 April 2017	11	Detected spawning, kelted, redd observed
I28	Ma	76	Tumwater	20 April 2017	2	Entered unmonitored tributary, shed tag mortality
I25	Fe	79	Tumwater	11 May 17	6	Detected digging, fallback below dam
I30(F4)	Fe	72	Enclosure	3 May 2017	8	Detected spawning, kelted
I32(M4)	Ma	75	Enclosure	3 May 17	5	Active 3 days, shed tag/mortality

**Figure 7:**
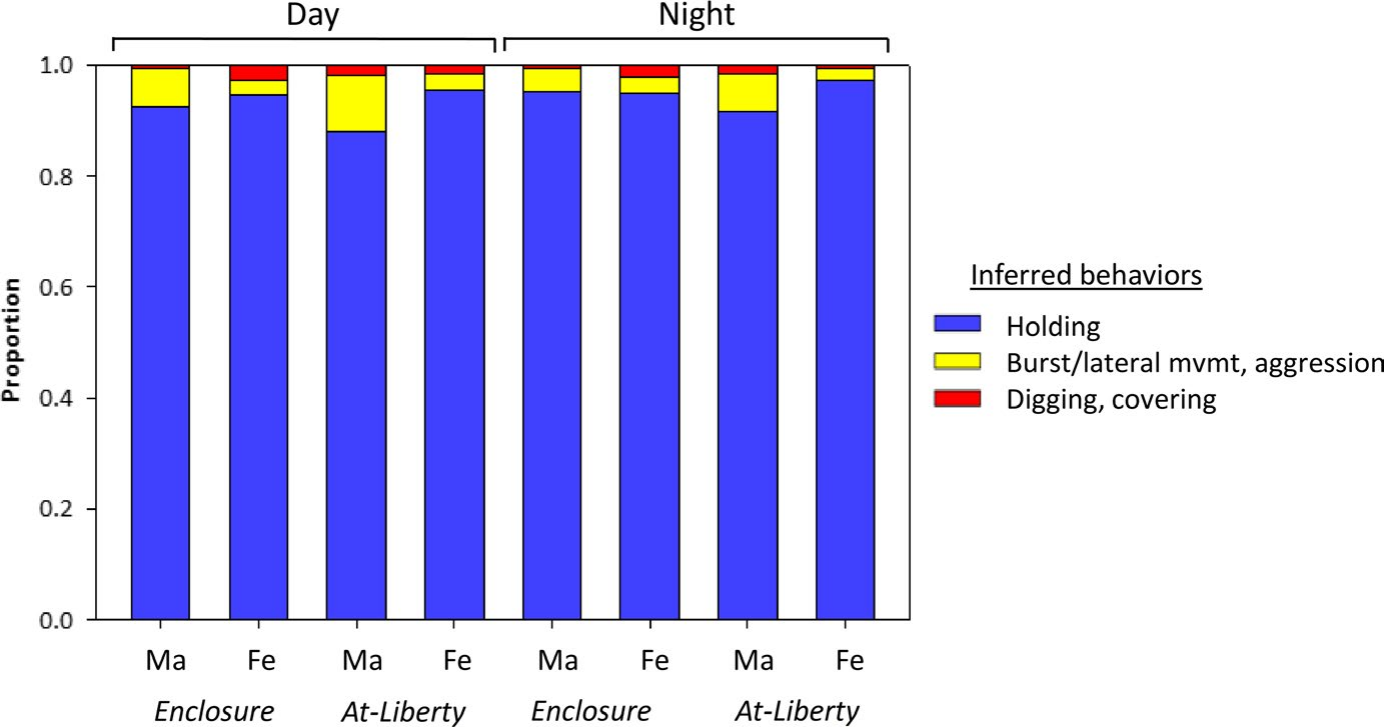
Relative time budget for inferred behaviors comparing enclosure and *at‐liberty* steelhead. Detections are split by sex, monitoring group (enclosure/*at‐liberty*), and by the time of day detections took place. Behaviors were classified using criteria in Figure 6. Male behaviors shown in red represent detected acceleration ≥0.90 g threshold and correspond to burst movement and aggressive behaviors

Acceleration records implied *at‐liberty* steelhead spent the majority of their time holding (Figure 7). The mean proportion holding for all *at‐liberty* steelhead was 94.6% and was slightly higher in females (96.5%) than males (89.9%). A mean of 1.7% of acceleration records was classified as burst movement/aggression (≥0.90 g) and 8.5% burst/lateral movement (0.21–0.90 g). The three female steelhead exhibiting oviposition/covering detections had nearly identical proportions of inferred behaviors (~97% holding, ~1.5% burst/lateral movements, ~1.5% digging, oviposition/covering), and these proportions were similar to those of the two females not detected ovipositing. Time budgets for enclosure and *at‐liberty* steelhead were qualitatively similar within sex, and there were not large differences in activity of *at‐liberty* and enclosure steelhead during daylight hours (~06:00 a.m. to 8:00 p.m.) versus at night (~08:00 p.m. to 06:00 a.m.; Figure 7).

## 4 DISCUSSION

We used accelerometer biotelemetry to infer spawning of *at‐liberty* female steelhead, quantify oviposition rate, and develop time budgets remotely using intragastrically implanted tags. A growing body of research has been conducted using accelerometer tags for monitoring of animal behaviors, but to date, most studies have used externally attached tags with limited monitoring duration (Broell et al., 2016), and many require recapture of archiving tags (Lowe, Holland, & Wolcott, 1998; Thiem et al., 2015). Intragastrically and surgically implanted accelerometer telemetry tag studies have been limited to studies of large‐bodied species (Moser et al., 2018; Whitney, Papastamatiou, Holland, & Lowe, 2007) capable of bearing larger tags. Such tags are especially useful in habitats or environmental conditions that often prevent direct observation of behavior (high river velocity/turbidity) and/or movements such as the large spatial distances between release location and spawning grounds in this study, which can preclude recovery of archival tags. To our knowledge, this may be the first use of accelerometer tags in fish that both transmitted acceleration data and that were nonsurgically inserted. Regardless of tag technology and attachment method, the classification and analysis framework presented here can be adapted for use in other systems as accelerometer tags are applied more widely.

A key element of the study was the direct observation of individuals bearing tags prior to releasing *at‐liberty* individuals. An important assumption is that observed behaviors are representative of those in the natural environment. It is possible that acceleration records of *at‐liberty* steelhead included behaviors not observed in the enclosures or behaviors that were misclassified because *at‐liberty* individuals had a wider behavioral repertoire. For example, fish monitored within enclosures may have exhibited more aggressive interactions due to the confined space. While our small sample precluded statistical comparisons, the qualitatively similar time budgets observed for enclosed and *at‐liberty* monitored steelhead, distinctiveness of key behaviors (e.g., covering), and similarity in behavior of enclosed steelhead compared to in situ observations of other salmonids during spawning (Esteve, 2005; Newcombe & Hartman, 1980) suggest any such bias in our study system to be minimal. Nonetheless, future studies should carefully design enclosure observations to minimize the potential for artifacts and for misclassification of key behaviors and strive to directly observe behaviors and validate telemetry inferences in *at‐liberty* animals when conditions allow.

Handling and tagging effects are a concern in any telemetry study, and the accelerometer tags provided evidence of post‐release short‐term changes in behavior. Steelhead in the enclosure exhibited 2–3 hr of high activity on the first day not displayed by the *at‐liberty* released steelhead, possibly in response to confinement. Nonetheless, the short‐term effects may have gone unrecognized in a traditional radio telemetry study and future use of accelerometer tags may identify the nature and duration of short‐term handling/tagging effects. Our design was not able to separate handling from tagging effects because we were unable to observe untagged controls and untagged control groups are recommended when possible.

The classification developed here demonstrates the importance of both the magnitude of acceleration and temporal variance in acceleration for identifying behaviors. For example, holding was distinguishable from magnitude alone, while both magnitude and frequency were necessary to distinguish between oviposition/covering and digging. Oviposition/covering was recognizable given high frequency, high magnitude detections over a consistent time period, whereas digging involved lower frequency, irregularly spaced high magnitude events. A limitation to our analysis was the criterion we used to distinguish oviposition from digging/aggression was based on a manual assessment of frequency of high g events, using the criteria in Figure 6. We do not think manual review severely biased the quantification of oviposition in our study, but we note that automated coding would be needed with larger datasets. While beyond the scope of our study, the future application of multivariate time series analyses or machine learning could further enhance the discriminatory power and accuracy of behavioral classifications based on acceleration time series data, and reduce costs and time spent on data processing and analysis.

We were not able to distinguish among all observed behaviors using the acceleration data produced by the nonarchival prototype tags that integrated the *x*‐*y*‐*z* acceleration axes into a single maximum value. Current archival tags can record three axes and future telemetry tags will transmit acceleration for each axis separately, increasing potential for discrimination. For example, qualitative laboratory testing revealed that mimicking the roll associated with a female digging by rotating the tag 90° would result in a burst reading reaching the max acceleration magnitude (1.5 g) in >50% of trials. Thus, we hypothesize digging and oviposition could be distinguished from aggression events using a 2‐ or 3‐axis tag because aggression events rarely involve rolling of the body. Similarly, changes in pitch would likely allow detection of probing (Esteve, 2005; Tautz & Groot, 1975) and more direct detection of oviposition in female steelhead. More broadly, pitch and/or roll are associated with feeding events in a wide variety of taxa.

We note that tag design and telemetry studies often require trade‐offs and consideration of key behaviors and acceleration forces during the design stage should help inform tag selection and specifications. For example, key behaviors may be readily identified with a single axis accelerometer, allowing smaller tag size, increased battery life, and/or increased temporal resolution. Increases in the number of transmitted data types (e.g., 1‐ vs. 3‐axis datasets) reduce the number of individual tag codes that can be programmed on a single frequency. Orientation of accelerometer(s) within the tag and tag placement on the animal will also affect the resulting acceleration data and classification criteria. The movement behaviors of target species require consideration because nonarchival tags require that receivers be positioned near the tagged subject species, which may not be feasible in every case. Monitoring of highly mobile species would likely require mobile rather than fixed‐array monitoring and would potentially limit the number of individuals monitored.

Assignment accuracy of acceleration records was high in most cases, but not error‐free. Digging behavior was detected using telemetry and was correctly assigned for 93% of events independently identified by video observation. The duration of digging events observed in the video and length of the BI accounted for some of the variability in recorded accelerations during digging events (0.27–1.5 g). Tags reported maximum acceleration every 4 s, and some digging events were recorded across two separate BIs. In these cases, the digging event acceleration records typically included one high magnitude record followed by a second record that was sometimes below the 0.9 g threshold (or vice versa), highlighting the potential role of sampling frequency (BI) on acceleration record classification. Importantly, such effects could introduce bias depending on the details of the classification protocol. For example, in cases where the event spanned two BIs and one record was below 0.9 g, the duration of digging would have been underestimated and the number of digging events overestimated if the adjacent records were independently classified. Secondary criteria such as reduced thresholds for adjacent records when estimating duration or requiring a lag before scoring a second event can eliminate or reduce such bias.

### 4.1 Steelhead spawning behaviors

**Figure 8:**
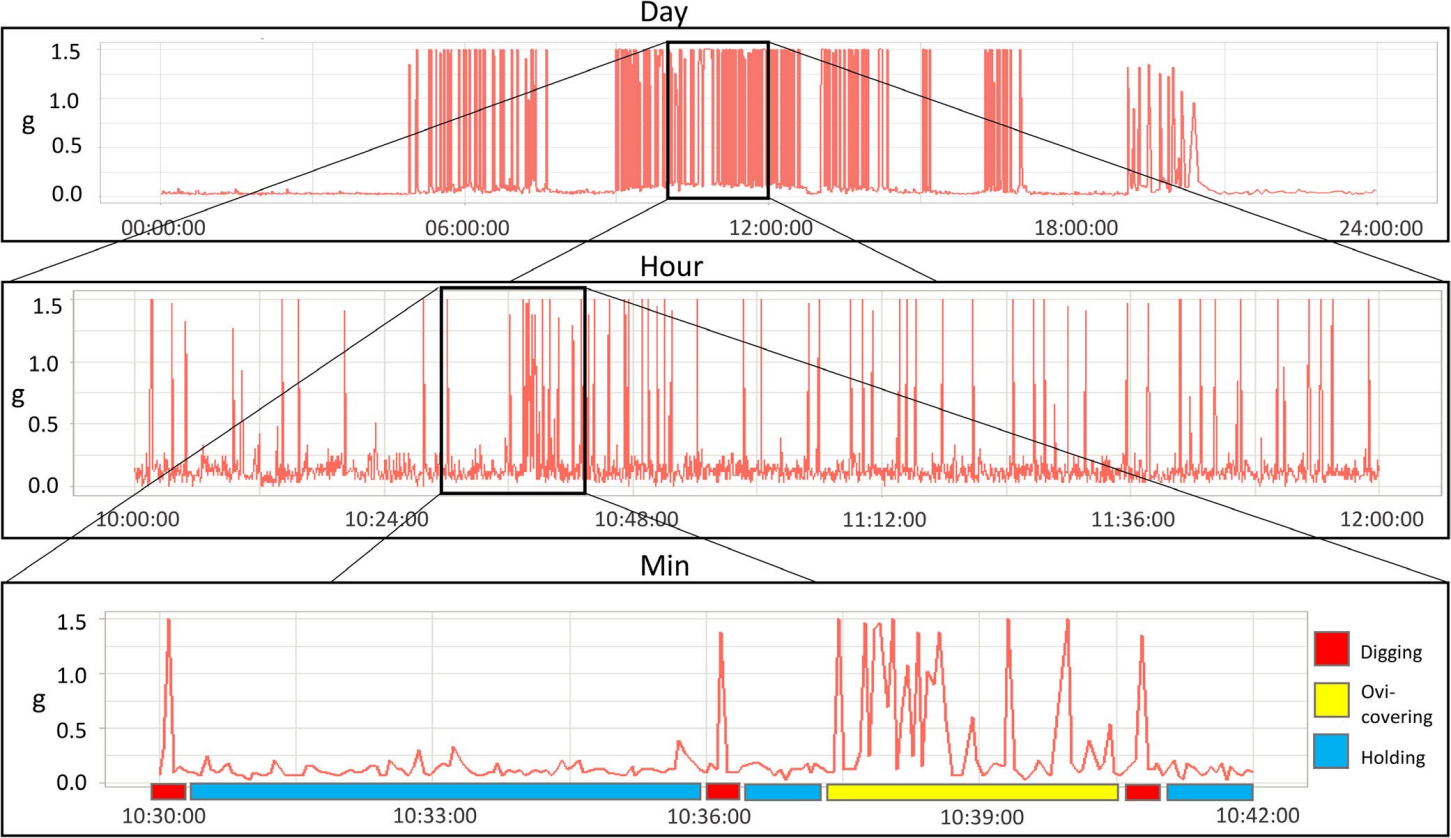
Example time series of acceleration for an *at‐liberty* steelhead, including an inferred oviposition event. Acceleration (g) per minute over a single day of detections (top bar), a two‐hour detection time series (middle bar), and 12‐min time series (bottom bar, showing all records). Inferred behaviors digging (red), oviposition/covering (yellow), and holding (blue) are indicated by bottom colored bar

The *at‐liberty* observations revealed patterns not obtainable from video observations. Oviposition events were uncommon during daylight video observations (video was unusable after dusk), and steelhead have been previously reported ovipositing at night (Needham & Taft, 1934). Interestingly, only one nocturnal (8:00 p.m. to 06:00 a.m.) oviposition/covering event was detected among all enclosure and *at‐liberty* steelhead suggesting nocturnal spawning activity may vary among populations or across environmental conditions. Similarly, prolonged holding (e.g., Figure 8) differed among females, suggesting intrinsic interindividual differences in diel behavior (e.g., related to behavioral syndromes) or experienced external stimuli.

A key objective was to estimate oviposition rate because redd counts are commonly used to estimate salmonid escapement and recruitment (Beland, 1996; Rieman & Myers, 1997) but the presence of test digs (false redds; Crisp & Carling, 1989) and detection error (missed redds) may introduce considerable bias in estimates (Dunham, Rieman, & Davis, 2001; Gallagher & Gallagher, 2005). Multiple redds/female have been previously reported in spawning enclosure experiments and in the wild (Berejikian, Doornik, LaRae, Tezak, & Lee, 2005; Kuligowski, Ford, & Berejikian, 2005; Orcutt et al., 1968). While we observed multiple oviposition events per female in our small sample, no female constructed more than one redd. Acceleration data records alone cannot be used to infer the presence of multiple redds without precise location information, but it does allow for the inference of a given females spawning rate and a maximum estimate of redds. The approach used here could be used to improve accuracy by quantifying the number of oviposition events/redds per female and assist in distinguishing test digs from true redds.

The spatial distribution of tagged animals will affect the ability to track movements and quantify behavior in natural settings. In this study, *at‐liberty* steelhead were challenging to monitor while migrating, resulting in some incomplete or short‐duration time series. While most individuals remained in spawning reaches for ~7 days, *at‐liberty* males were particularly challenging to monitor because they rarely remained within 500 m reaches for longer than a single day. Nonetheless, records from two *at‐liberty* males were consistent with enclosure observations and past research, and suggested males increase access to females by ranging over a larger territory during spawning and that aggressive interactions among males are common (Esteve, 2005; Foote, 1990; Kuligowski et al., 2005).

Accelerometers can be used to quantify detailed time budgets for individual animals at multiple scales and applied to analyses of mating behavior, energetics, foraging, and antipredator behaviors. Time budgets were generally similar between enclosure and *at‐liberty* steelhead, and revealed holding was a large majority of the time budget, consistent with a capital breeding species, and that most mating activity occurred during daytime. For example, *at‐liberty* female steelhead were more commonly detected making movements >0.0 g during daylight hours (mean 74% recorded during day vs. 26% after dark).

### 4.2 Future considerations

Ongoing advances in telemetry technology will continue to improve the sophistication and reduce the size of telemetry tags, opening future research potential provided telemetry data can reliably be linked to specific behaviors. In our study, we provide a framework for identifying and quantifying specific behaviors using remote accelerometer telemetry without surgical procedures. Future applications of gastric accelerometer tags in anadromous fishes could include (a) monitoring for differences in spawning behavior and success between hatchery‐reared and natural‐origin adults; (b) detection of spawning behaviors and distinguishing between holding versus spawning habitats in systems with periodic or chronic high turbidity/poor visibility (i.e., glacial melt fed streams); and (c) evaluating energy costs and swimming performance thresholds affecting passage success at migration obstacles, including fishways. Other potential applications include quantification of foraging and mating behaviors, though this would require surgical or external attachment of tags.

Beyond fishes, acceleration sensory tags may be applied to a wide range of taxa and research questions. A similar framework to that used here could quantify behavior in habitats where direct observation of animals in situ is limited or impossible, including in nocturnal species, aquatic species living in turbid waters, or terrestrial species inhabiting thick vegetation. Regardless of habitat, the technology will allow rapid quantification of behaviors in great detail and sample sizes adequate to permit population‐level inferences, assuming receiver arrays or mobile tracking can adequately monitor tag outputs. For example, foraging behavior studies would benefit greatly from detailed data provided by movement sensor tags that monitor head movements (Kokubun, Kim, Shin, Naito, & Takahashi, 2011; Laich et al., 2008). Similarly, habitat association studies can be greatly refined by linking specific behaviors to location data acquired from telemetry (Jessopp, Cronin, & Hart, 2013; Wakefield et al., 2009). Coupling interindividual mating activities with pedigree analysis could provide important insights into the links between mating behavior, phenotype at the time of tagging, and individual fitness. Use of tags to identify timing and habitats of key behaviors such as mating or spawning may be especially helpful in the management of invasive or nuisance species by pinpointing areas for control effort. While archival sensor technology has been applied to such studies in the past (e.g., Halsey, Shepard, & Wilson, 2011; Wilson, Hinch, Eliason, Farrell, & Cooke, 2013), the advent of real‐time accelerometry via radio telemetry will allow application to a much broader range of species of intellectual, economic, or conservation importance. Regardless of future applications and advancing technology, we advocate the direct observation of telemetered animals to establish criteria for the recognition of key behaviors prior to inferences about behavior of *at‐liberty* animals monitored via accelerometer biotelemetry, combined with a systematic classification approach for the analysis of acceleration time series.

## CONFLICT OF INTEREST

None declared.

## AUTHORS CONTRIBUTIONS

Caudill and Fuchs conceived and designed the study; Fuchs conducted fieldwork; Fuchs and Caudill analyzed data; Fuchs drafted the manuscript; Fuchs and Caudill substantially contributed to the writing; and Caudill secured funding and administered the project and gave final approval for publication.

## Data Availability

All data will be available through “University of Idaho Northwest Knowledge Network (NKN).” At DOI url: https://doi.org/10.7923/03xb-ej79

## APPENDIX 1

Assigned observed behavioral counts and proportions.

**Table A1:** Mean acceleration (g) by individual steelhead and behavior during enclosure observations

Mean acceleration of assigned behaviors (g)									
Fish No.	Holding	Burst movement	Lateral movement	Digging	Oviposition	Covering	Coaxing	Aggression	Out of view
Females									
F1	.06	.46	.24	1.26	.18	1.20	–	.55	.13
F2	.12	.33	.25	1.25	.32	1.15	–	.55	.12
F3	.13	.38	.30	1.34	.19	1.01	–	–	.16
Mean	.10	.37	.27	1.27	.24	1.14	–	.55	.13
Males									
M1	.08	.30	.24	–	–	–	.40	.37	.10
M2	.09	.39	.30	–	–	–	.36	.54	.12
M3	.07	.39	.26	–	–	–	.32	.64	.14
Mean	.08	.36	.27	–	–	–	.37	.51	.12
Mean total	.09	.37	.27	1.27	.24	1.14	.37	.51	.12

Note

Behaviors were identified using video observations.

**Table A2:** Proportions of total video‐assigned behaviors for steelhead monitored within enclosure by individual steelhead

Proportion of assigned behaviors									
Fish No.	Holding	Burst moves	Lateral moves	Digging	Oviposition	Covering	Coaxing	Aggression	Out of view
Females									
F1	.566	.034	.034	.030	.001	.003	–	.002	.331
F2	.697	.018	.025	.035	.001	.008	–	.005	.211
F3	.654	.036	.039	.026	.001	.005	–	.000	.239
Mean	.639	.029	.032	.030	.001	.006	–	.002	.260
Males									
M1	.647	.026	.036	–	–	–	.027	.011	.253
M2	.525	.033	.063	–	–	–	.057	.040	.282
M3	.715	.022	.031	–	–	–	.003	.003	.226
Mean	.629	.027	.043	–	–	–	.029	.018	.253
Mean total	.634	.028	.038	.030	.001	.006	.029	.010	.257

**Table A3:** Results of Dunn's test following nonsignificant *p*‐values are indicated in bold

	Behaviors							
	Out of view	Holding	Burst moves	Lateral moves	Aggression	Coaxing	Oviposition	Covering
Holding	.000	–	–	–	–	–	–	–
Burst moves	.000	.000	–	–	–	–	–	–
Lateral moves	.000	.000	**.061**	–	–	–	–	–
Aggression	.000	.000	.037	.000	–	–	–	–
Coaxing	.000	.000	**.210**	.004	**.367**	–	–	–
Oviposition	.000	.000	**.255**	**.409**	**.105**	**.168**	–	–
Covering	.000	.000	.012	.001	**.220**	**.070**	.039	–
Digging	.000	.000	.000	.000	.030	.001	.027	**.922**

Note

All values represent adjusted *p*‐values, and nonsignificant *p*‐values are indicated in bold.
